# Experimental Climate Warming Reduces Floral Resources and Alters Insect Visitation and Wildflower Seed Set in a Cereal Agro-Ecosystem

**DOI:** 10.3389/fpls.2022.826205

**Published:** 2022-02-23

**Authors:** Ellen D. Moss, Darren M. Evans

**Affiliations:** ^1^School of Natural and Environmental Sciences, Newcastle University, Newcastle Upon Tyne, United Kingdom; ^2^School of Biological, Biomedical and Environmental Sciences, University of Hull, Hull, United Kingdom

**Keywords:** climate change, ecological network, ecosystem service, field experiment, pollination, simulation, species interaction, wildflower seed set

## Abstract

Declines in pollinating insects and wildflowers have been well documented in recent years. Climate change is an emerging threat to insect pollinators and their food plants, but little is known about how whole communities of interacting species will be affected or what impacts there may be on ecosystem services such as pollination. Using a novel open-air field experiment, we simulated an increase in temperature of 1.5°C and rainwater of 40% for two growing seasons to investigate how climate change may impact several within-field features of temperate arable agro-ecosystems: (1) wildflower floral resources; (2) insect visitation; (3) flower-visitor network structure; and (4) wildflower seed set. Experimental warming reduced total floral abundance by nearly 40%, and nectar volumes by over 60% for two species. The species richness of the visiting insects and flowering plants (dominated by annuals) were unaffected by warming, and while a negative impact on visitor abundance was observed, this effect appears to have been mediated by different community compositions between years. Warming increased the frequency of visits to flowers and the complexity of the flower-visitor interaction networks. Wildflower seed set was reduced in terms of seed number and/or weight in four of the five species examined. Increased rainwater did not ameliorate any of these effects. These findings demonstrate the adverse impacts that climate warming might have on annual wildflowers in arable systems and the pollinating insects that feed on them, highlighting several mechanisms that could drive changes in community composition over time. The results also reveal how cascading impacts within communities can accumulate to affect ecosystem functioning.

## Introduction

Recent declines in pollinator species diversity and abundance are a major global concern given their importance to human nutrition, economics, ecosystems, and agriculture (IPBES, 2016). These declines have been attributed to a number of factors such as agricultural intensification, land use change, and disease (Settele et al., 2016; Dicks et al., 2021). With global temperatures expected to rise by at least 2°C by the end of the twenty-first century (relative to 1850–1900) (IPCC, 2021), climate-warming is expected to compound these pollinator declines by causing range shifts and phenological changes, with some recent evidence for bumblebee convergence across continents (Kerr et al., 2015). There is particular concern regarding how changing phenologies and distributions of plants and their pollinators may lead to temporal and spatial mismatches between them (Gérard et al., 2020). However, there are currently too few empirical studies to draw conclusions about the direct and indirect impacts of climate change on plant-pollinator communities and interactions (Scaven and Rafferty, 2013; Settele et al., 2016), or what these impacts will mean for ecosystem functioning and human society (Dicks et al., 2021).

To date, most studies investigating the effects of climate change on pollinators have examined individual species or a subset of wild pollinators (Parmesan, 2006), which has demonstrated shifts in both spatial (altitudinal and latitudinal) (Parmesan, 2006; Kerr et al., 2015; Pyke et al., 2016) and temporal distributions (Bartomeus et al., 2011; Hassall et al., 2017). However, a more fundamental challenge is to understand how climate change will affect entire communities of interacting plants and animals to gain a better understanding not only of biodiversity responses, but also the impacts on ecological processes such as pollination (Settele et al., 2016). Species-interaction networks are well suited to investigating such questions as they characterise community structure and complexity, allowing the assessment of how many small changes at the species level can add up to significant community-scale impacts (Scaven and Rafferty, 2013). However, few studies have used ecological networks to examine community responses to climate-warming. Memmott et al. (2007) computationally simulated climate-change driven phenological mismatches and found they lead to extinctions of both plants and pollinators. Evidence from long-term observational datasets of plant-pollinator interactions have demonstrated temporal and spatial mismatches and changes in network structure (Burkle et al., 2013), though an experimental study manipulating flowering onset found no evidence of temporal mismatching (Rafferty and Ives, 2011). Research employing a latitudinal climate-gradient found that temperature and precipitation were important drivers of structural network properties that are shaped by species richness and phenology (Petanidou et al., 2018). A recent modelling study found that pollinator phenology was an important determinant of network robustness and plant species persistence (Ramos-Jiliberto et al., 2018). The approaches used in these studies have yielded valuable insights into how climatic changes may affect plant-pollinator networks, but so far there have been no experiments, to our knowledge, simulating climate-warming in natural field conditions to investigate this topic.

An increasing body of research has looked at how pollinator loss affects pollination and ecosystem functioning, with studies finding links between pollinator visitation and wildflower seed set (Franzén and Larsson, 2009; Lundgren et al., 2016), crop yield (Garibaldi et al., 2013), and seedling diversity (Lundgren et al., 2016). However, very few studies have investigated this in the context of climate change and those that have, usually focused on just one plant species. Kudo and Cooper (2019) demonstrated that a wildflower and its pollinators showed differing sensitivity to early onset of spring, which caused phenological mismatches, and this in turn led to reduced seed set. Thomson (2010) also observed increasing asynchrony and pollination limitation between an early sub-alpine plant and its bumblebee pollinator. Burkle et al. (2013) quantified the pollen grains carried by bees caught over a 120 year period, which showed a decline in pollination service. Bishop et al. (2016) demonstrated that insect pollinators were able to recover yield losses in faba bean (*Vicia faba*) plants after moderate heat stress, suggesting that increased visitation could offset some of the negative effects for this species. Therefore, although climate change is likely to result in phenological mismatches and potentially disrupt pollination, predicting the impacts on plant-pollinator communities is hampered by a lack of field experimentation and community-scale investigations.

We established a simulated climate-warming experiment to examine the impacts of elevated temperature and increased rainwater on flowering plants and flower-visiting insects. Temperature was increased by 1.5°C, which aligns with current climate projections for Northern Europe (IPCC, 2021), while precipitation was increased by 40%. Summers in the United Kingdom have been 15% wetter on average over the last decade than during the 1981–1990 period, and over 17% wetter than the 1961–1990 period (Kendon et al., 2021). Given that rainfall patterns across the United Kingdom are predicted to substantially vary on seasonal and regional scales in the future (Met Office, 2021), our intention here was twofold: (1) simulate a situation where rainfall was well above the seasonal norm and (2) investigate whether increasing rainwater as an irrigation option in a warming world would offset the effects of raised temperature. The experimental method uses a bottom-up approach, whereby only the lowest trophic level is manipulated directly, but resultant changes in insect visitation could be observed in addition to the plant responses (Scherber et al., 2010). Our experiment had four main objectives: (1) Investigate how experimental warming affects floral resources. We predicted these resources would be negatively affected, as increases in temperature can lead to reductions in both the number of individual plants, flowers per plant, and nectar volumes (Liu et al., 2012; Mu et al., 2015; Takkis et al., 2018; Borghi et al., 2019). (2) Observe any changes in insect visitation. Given the bottom-up approach and open-air nature of the experiment, we expected to observe indirect impacts on the flower-visitor community via foraging behaviour (Gérard et al., 2020), as floral abundance can positively affect insect visitation (Fowler et al., 2016). (3) Examine the impacts of warming on flower-visitor network structure. We predicted that network structure would be affected by reductions in floral resources, as structure can be altered by changes in food resources and pollinator behaviour (Scaven and Rafferty, 2013), and by changes in phenology (Burkle et al., 2013; Petanidou et al., 2018). (4) Investigate how experimental warming affects wildflower seed set. We predicted negative impacts on seed production, as increasing temperatures can directly reduce seed set in non-crop (Jin et al., 2011) and crop plants (Liu et al., 2016), and indirectly reduce seed set via pollinator phenological mismatch (Kudo and Cooper, 2019), although it is possible this could be offset by increased insect visitation (Bishop et al., 2016; Lundgren et al., 2016).

## Materials and Methods

### Experimental Approach

The experiment was conducted at Stockbridge Technology Centre (53°49′N–1°9′W) in North Yorkshire (United Kingdom), an arable farm growing crops both commercially and for agricultural research. Our experimental setup replicated that of Rollinson and Kaye (2012). The experiment consisted of 24 outdoor 2 × 2 m plots in an agricultural field, separated by 2 m buffers, in a randomised block design with 6 replicates of four treatments: 1.5°C increase in temperature above ambient (“Heat”); 40% increase in precipitation (“Water”); warming and precipitation treatments combined (“Heat + Water”); and ambient conditions (“Control”) (Supplementary Figure 1). The heated plots were warmed with non-convective infrared heaters (model: MSR-2420, Kalglo Electronics Inc., Bethlehem, PA, United States) suspended 1.5 m above them, operating continuously from the date of assembly (16/04/14 and 15/04/15) until end of sampling (19/08/14 and 18/08/2015). These infra-red heaters warm soil and vegetation surfaces directly, similar to solar radiation, rather than air (Kimball, 2005). Temperature differences between heated and unheated plots were maintained at a consistent level using a real-time proportional-integrative-derivative feedback system that connected to the heaters, to a data logger (Campbell Scientific; Loughborough, United Kingdom), and to six infrared temperature sensors (IR120; Campbell Scientific; Loughborough, United Kingdom) measuring surface temperatures in the plots every 10 s. The feedback system switched the heaters on/off as needed to maintain the required temperature difference between the heated and unheated plots. The temperature sensors were randomly assigned to an unheated and heated plot within each block (see Supplementary Figure 1), where they pointed at the centre of the plots and were sited 1.1 m above them. “Dummy” heaters were suspended above unheated plots to mimic any potential structural effects. The precipitation increase was simulated by distributing collected rainwater using a watering can; volumes were based on mean monthly rainfall data collected between 2002 and 2011 at the farm’s weather station (13L in April, 19L in May, 24L in June, 26L in July, and 30L in August). While the temperature increase was targeted at 2°C, a mean increase of 1.5°C was actually achieved during the experiment. *In situ* active-warming methods are the most precise and consistent methods of experimental warming (Ettinger et al., 2019) and the present setup is an effective and economically viable approach (Kimball, 2005; Rollinson and Kaye, 2012; Derocles et al., 2018).

Prior to equipment assembly, the plots and buffers were sown with spring wheat (*Triticum aestivum* cultivar Tybalt) and the plots were additionally sown with an arable wildflower seed mixture using quantities that are appropriate for establishing a grass/wildflower meadow in 4 m^2^ (see Supplementary Table 1 for species and sowing information). Eight wildflower species were selected based on several criteria: insect-pollinated, native to the United Kingdom (or naturalised historic introductions from continental Europe), annual, found in arable fields, and able to grow in a within-crop habitat (Fitter and Peat, 1994; Rose and O’Reilly, 2006). No pesticides were applied to the plots after sowing. Invasive non-crop plants were controlled by hand weeding each plot for 10 min each week until the wheat and wildflowers had established, but non-sown flowering species were allowed to grow and flower.

We describe specific data collection and generation methods under each objective below. Sampling took place between the start of flowering in early June and the end of August (i.e., harvest) in 2014 and 2015. Seven sampling rounds were conducted in each year, spanning the entire flowering period, with the dates matched as closely as possible to ensure even sampling between years.

### Objective 1: Wildflower Floral Resources

All flowering plant species were identified, and all floral units counted, in each plot during each sampling round. Floral abundance and flowering plant richness for each plot were summed across sampling rounds to give totals for the whole season (it is possible that some flowers may have been counted twice, but unlikely because repeat surveys of plots were usually at least a week apart). In mid-June 2015, five flower buds in each plot from three early flowering species (*Lamium purpureum, Stellaria media*, and *Veronica persica*) were enclosed within small fine-mesh drawstring bags and nectar volume was sampled using 0.5 μL microcapillary tubes once the flowers had opened (Kearns and Inouye, 1993). This process was repeated in late July 2015 for two later-flowering species (*Centaurea cyanus* and *Glebionis segetum*). Nectar was unobtainable from *S. media* and *G. segetum* due to the nectaries being too small for the microcapillary tubes available.

### Objective 2: Flower Visitation

Each plot was observed for a total of 20 min per sampling round, during which, insect specimens feeding from flowers were captured using a hand-net and euthanised with ethyl acetate in individual tubes. All insect samples were identified to species level, or as close to as possible, by taxonomists using morphological keys (see Supplementary Material for a list). Sampling took place between 9:00 and 17:00 and during appropriate weather: temperatures of at least 15°C, no more than a slight wind, and no precipitation. Insect visitor abundance and richness values were pooled across sampling rounds. Species accumulation curves were created for each plot to examine sampling completeness of insect visitors. Asymptotes were not reached so species richness was extrapolated and Chao estimates (Chao, 1987) of richness calculated using the “vegan” R package (Oksanen et al., 2020). Diet breadth was calculated across all visitor species visiting each plot, as the mean number of plant species each pollinator species visits. Frequency of visits to all flowers was calculated for each plot (visits/flowers), and for two of the sown species that had sufficient data: *G. segetum* and *C. cyanus*.

### Objective 3: Flower-Visitor Networks

A species interaction network was constructed for each plot and network descriptors calculated using the “networklevel” function of the “bipartite” package in R (Dormann et al., 2008). Four quantitative network metrics (Bersier et al., 2002) appropriate for mutualistic networks were chosen to examine changes in network complexity, consumer-resource asymmetries, and evenness of structure:

• Weighted Connectance (C_q_): the number of potential interactions that are realised.
• Generality (G_q_): the number of flower species per visitor species.
• Vulnerability (V_q_): the number of visitor species per flower species.
• Interaction Evenness: how even the frequency of the different interactions is.

### Objective 4: Wildflower Seed Set

Seed heads of three sown wildflower species (*C. cyanus*, *G. segetum*, and *L. purpureum*) and two resident species (*V. persica* and *S. media*) were collected for analysis. Collection for each species occurred once there were at least 10 ripe seed heads present in all the plots (the early spring species (*L. purpureum, V. persica*, and *S. media*) were not sampled in 2014 due to logistical constraints). Each collection involved randomly selecting 5 ripe seed heads from each plot, which were dried in an oven at 80°C for 48 h to control for any weight differences due to water content. Seed heads were processed individually: seeds were counted, a dry-weight measurement of all seeds was taken, and average seed weight calculated (mg). There were two sampling events for *G. segetum* in both years, and for *C. cyanus* in 2014, to account for the prolonged flowering periods observed in these two species.

### Data Analysis

All datasets were analysed with regression models, using R version 4.1.0 (R Core Team, 2021) and the “lme4” (Bates et al., 2015) and “glmmTMB” (Brooks et al., 2017) packages. Selection of distribution families was based upon the type of data to be analysed (e.g., Poisson and negative binomial for count data, Gaussian for decimal, gamma and inverse gaussian for positive decimal, beta for decimal bounded by 0 and 1) and on model validation assessments using the “DHARMa” package (Hartig, 2021). Data were transformed when all distribution and link options produced poor fitting models (Table 1). See Supplementary Material for further details of the modelling process.

**TABLE 1:** Effect of treatment and year on all plant and insect-visitor response variables (d.f = 1 for year, 3 for treatment, 3 for treatment:year interaction).

Response variable	*n*	Treatment		Year		Interaction	
				
		Statistic	*p*	Statistic	*p*	Statistic	*p*
**(1) Floral resources**							
Plant species richness	48	(LRT) 4.934	0.177	(LRT) 51.069	< *0.001*	(LRT) 3.205	0.361
Total floral abundance	48	(LRT) 20.378	*0.001*	(LRT) 9.431	*0.002*	(Dev) 2.027	0.567
*C. cyanus* nectar volume*	61	(LRT) 4.539	0.209	-	-	-	-
*L. purpureum* nectar volume	34	(F) 6.251	*0.002*	-	-	-	-
*V. persica* nectar volume	39	(LRT) 10.497	*0.015*	-	-	-	-
**(2) Visitation**							
Visitor species richness (extrapolated)	48	(F) 0.324	0.808	(F) 0.010	0.922	(F) 0.763	0.521
Visitor abundance	48	(Dev) 1.882	0.597	(Dev) 19.844	< *0.001*	(Dev) 10.576	*0.014*
Visits per flower	48	(F) 6.954	< *0.001*	(F) 0.878	0.354	(F) 1.515	0.225
Diet breadth	48	(F) 1.327	0.278	(F) 15.038	< *0.001*	(F)0.200	0.896
Visits per *C. cyanus* flower	48	(F) 1.010	0.399	(F) 1.566	0.218	(F) 1.115	0.356
Visits per *G. segetum* flower	48	(F) 4.415	*0.009*	(F) 0.132	0.718	(F) 1.488	0.232
**(3) Networks**							
Weighted connectance	48	(LRT) 13.118	*0.004*	(LRT) 18.625	< *0.001*	(LRT) 3.011	0.390
Generality	48	(F) 0.078	0.971	(F) 11.772	*0.001*	(F) 0.311	0.817
Vulnerability	48	(F) 0.211	0.888	(F) 0.749	0.392	(F) 0.274	0.844
Interaction evenness	48	(LRT) 9.743	0.021^†^	(LRT) 1.600	0.206	(LRT) 5.049	0.168
**(4) Seed set**							
*C. cyanus* seed number (2014)	240	(LRT) 30.125	< *0.001*	-	-	-	-
*C. cyanus* seed weight (2014)	240	(LRT) 10.895	*0.012*	-	-	-	-
*C. cyanus* seed number (2015)	55	(LRT) 5.683	0.128	-	-	-	-
*C. cyanus* seed weight (2015)	55	(LRT) 4.892	0.180	-	-	-	-
*G. segetum* seed number (2014)	138	(LRT) 9.186	*0.027*	-	-	-	-
*G. segetum* seed weight (2014)	138	(LRT) 15.543	*0.001*	-	-	-	-
*G. segetum* seed number (2015)	144	(LRT) 23.687	< *0.001*	-	-	-	-
*G. segetum* seed weight (2015)	144	(LRT) 18.093	< *0.001*	-	-	-	-
*L. purpureum* seed weight	110	(LRT) 15.962	*0.001*	-	-	-	-
*V. persica* seed number	119	(LRT) 29.646	< *0.001*	-	-	-	-
*V. persica* seed weight**	119	(LRT) 17.323	< *0.001*	-	-	-	-
*S. media* seed number	117	(LRT) 20.035	< *0.001*	-	-	-	-
*S. media* seed weight***	117	(LRT) 1.432	0.698	-	-	-	-

*Treatment and year p-values are derived from models without the interaction term (where present) unless it was significant. Significant p-values (p < 0.05) are italicised. Sample sizes, means, and regression coefficients for each treatment and year level are reported in Supplementary Tables 3–6. Test statistics vary between datasets due to the different models and distribution families that were employed (see Supplementary Material for further details).*

*Cube root (^1/3) transformed before analysis.

**Log transformed before analysis.

***Square (^2) transformed before analysis.

*^†^Non-significant at Bonferroni corrected alpha of 0.0125.*

The network, floral abundance, flowering-plant richness, and all of the flower-visiting insect datasets (abundance, richness, diet breadth, and visits per flower) were collected at the plot level (1 value per plot, per year). These datasets were analysed with generalised linear models (GLMs) including “treatment:year” interaction terms, where “treatment” was a factor with four levels (Control, Water, Heat, Heat + Water) and “year” a factor with two levels (2014 and 2015). Significance of the fixed effects were determined via two-way ANOVA, to assess the overall impact of all treatment levels (and both years) upon each variable. Where the interaction was non-significant, the models were re-run without it. Where the ANOVA revealed a significant effect of treatment, plots of the data and the regression coefficients and statistics from the GLMs were interrogated to determine which experimental treatments differed significantly from Control (and each other), including whether or not additional water could ameliorate any negative impacts that warming may have. A Bonferroni correction was applied to the network descriptor results to account for intercorrelation due to overlap in the different network properties that they are calculated from Tylianakis et al. (2007). Community dissimilarities for flowering plants and insects across the treatments were assessed using PERMANOVA via the “adonis2” function of the “vegan” package.

The nectar and seed datasets contain multiple values per plot and so they were analysed using mixed effects models, where treatment was a fixed effect (again as factor with four levels), and plot and collection date (for the *C. cyanus* and *G. segetum* models) were random effects (random intercepts). Significance of the fixed effect was determined via one-way ANOVA, and once again where this revealed a significant effect, differences between treatment levels were determined via plots of the data and the model regression coefficients and statistics. Two of the species of wildflower had multiple years of seed data available (*C. cyanus* and *G. segetum*), but these were analysed separately for each year due to uneven sampling and to maintain consistency in the analysis method across the dataset. *L. purpureum* seed number was not analysed as this species produces a maximum of only four seeds per seed head (Fitter and Peat, 1994) and it proved difficult to differentiate between those where seeds had fallen out and those where fewer had developed.

## Results

### Objective 1: Wildflower Floral Resources

**FIGURE 1:**
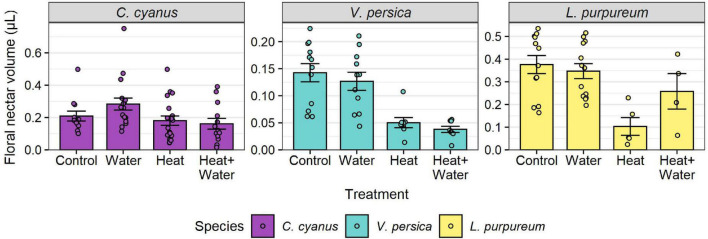
Raw nectar volumes per flower for each treatment, for three wildflower species. Bars and error-bars represent mean ± s.e. Points show the individual samples. Many flower buds were damaged or failed to open after being bagged, leading to uneven and smaller sample sizes than the target of 30 per treatment (see Supplementary Table 3).

**FIGURE 2:**
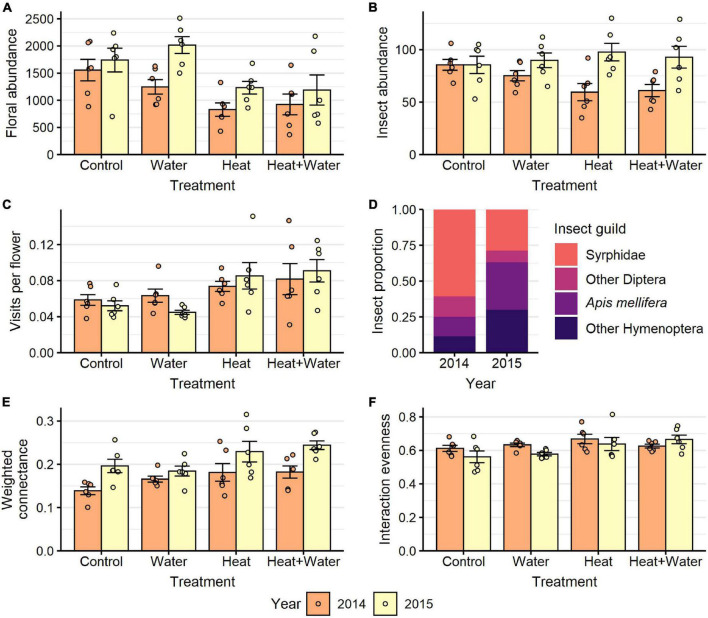
Responses to treatment in each year for: **(A)** floral abundance, **(B)** flower-visiting insect abundance, **(C)** visits per flower, **(E)** weighted connectance, **(F)** interaction evenness. **(D)** Shows the proportion of visitors belonging to different insect guilds in each year. Bars and error-bars represent mean ± s.e. Points show the individual samples. *n* = 6 per treatment per year.

A total of 27,326 flowers from 25 plant species were counted in 2014 and 37,066 flowers from 19 species in 2015. The most abundant flowers were those of *Glebionis segetum*, *Centaurea cyanus*, *Veronica persica*, *Capsella bursa-pastoris*, *Stellaria media*, and *Lamium purpureum*. There was no significant difference in the flowering-plant community composition or species richness between the treatments and no treatment:year interaction (Table 1 and Supplementary Table 7), but there was a significant difference between the 2 years (community: *F* = 20.549, *p* = 0.001; richness: LRT = 51.069, *p* < 0.001). Experimental warming significantly reduced the nectar volumes of *L. purpureum* and *V. persica*; volumes were 72.5 and 64.7% lower in the Heat treatment vs. Control, respectively, but *C. cyanus* was unaffected (Figure 1, Table 1, and Supplementary Table 3). There was a significant effect of treatment (LRT = 20.378, *p* < 0.001) and year (LRT = 9.431, *p* = 0.002) on total floral abundance, which was significantly lower in the two heated treatments compared to Control: 37.5 and 35.9% lower in the Heat and Heat + Water treatments respectively (Figure 2A and Supplementary Table 3). Increased precipitation had no significant effect on floral abundance (Figure 2A and Supplementary Table 3) or nectar volumes (Figure 1 and Supplementary Table 3) and did not offset the negative impacts of warming (except possibly for *L. purpureum* nectar (Figure 1 and Supplementary Table 3), though the sample size is very small).

### Objective 2: Flower Visitation

**FIGURE 3:**
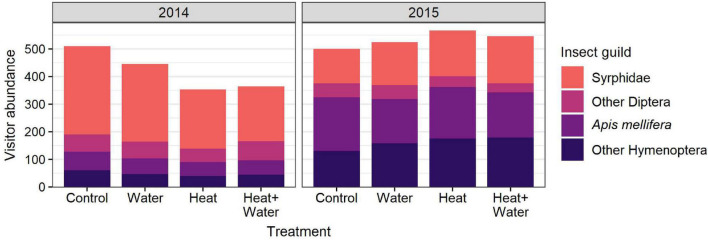
Total abundance of flower-visitors belonging to different insect guilds in each treatment, in each year.

A total of 1,687 flower visits from 80 insect species were recorded in 2014, and 2,195 flower visits from 69 species in 2015. The most abundant groups were hoverflies (Syrphidae), honeybees (*Apis mellifera*), bumblebees (*Bombus* sp.) and other non-syrphid Diptera. There was a significant difference in the insect community composition between the treatments (*F* = 4.031, *p* = 0.004) and the 2 years (*F* = 44.261, *p* = 0.001) but no interaction (Supplementary Table 7), with a notable shift from flies in 2014 to bees in 2015 (Figures 2D, 3). Conversely, extrapolated insect species richness was unaffected by treatment or year (Table 1). There was a significant treatment:year interaction for flower-visitor abundance (Deviance = 10.576, *p* = 0.014); in 2014 there were fewer visitors in both heated treatments relative to Control, while 2015 showed no such pattern (Figure 2B and Supplementary Table 4). The reduction in visitor abundance in the heated plots during 2014 appears to predominantly be caused by reductions in hoverfly abundance, while the other insect groups are less affected (Figure 3). The frequency of visits per flower for all species combined and for *G. segetum* (the species with highest floral abundance) were significantly increased by experimental warming (all species: *F* = 6.954, *p* < 0.001; *G. segetum*: *F* = 4.415, *p* = 0.009) (Figure 2C and Supplementary Table 4), but there was no effect of year (Table 1). The frequency of visits to *C. cyanus* flowers was unaffected by treatment or year (Table 1). Mean diet breadth of visitors was unaffected by treatment (Table 1) but there was a significant effect of year (*F* = 15.038, *p* < 0.001). Increased precipitation had no significant effects on flower visitation either in the presence or absence of warming (Figures 2, 3 and Supplementary Table 4).

### Objective 3: Flower-Visitor Networks

Weighted connectance was significantly increased under experimental warming (LRT = 13.118, *p* = 0.004) and there was also a significant effect of year (LRT = 18.625, *p* < 0.001) with higher values in the second year (Figure 2E and Supplementary Table 5). Interaction evenness was unaffected by year (Figure 2F and Table 1) and while it was initially shown to increase significantly under experimental warming (LRT = 9.743, *p* = 0.021), this effect was not significant after applying a Bonferroni correction (Table 1). Generality was unaffected by treatment (Table 1) but there was a significant effect of year (*F* = 11.772, *p* = 0.001) with higher values in 2014 (Supplementary Table 4). Vulnerability was unaffected by treatment or year (Table 1). The structure of the networks appears consistent across treatments, but very different between years (Supplementary Figure 2). Increased precipitation had no significant effects on the flower-visitor networks either in the presence or absence of warming (Figure 2 and Supplementary Table 5).

### Objective 4: Wildflower Seed Set

**FIGURE 4:**
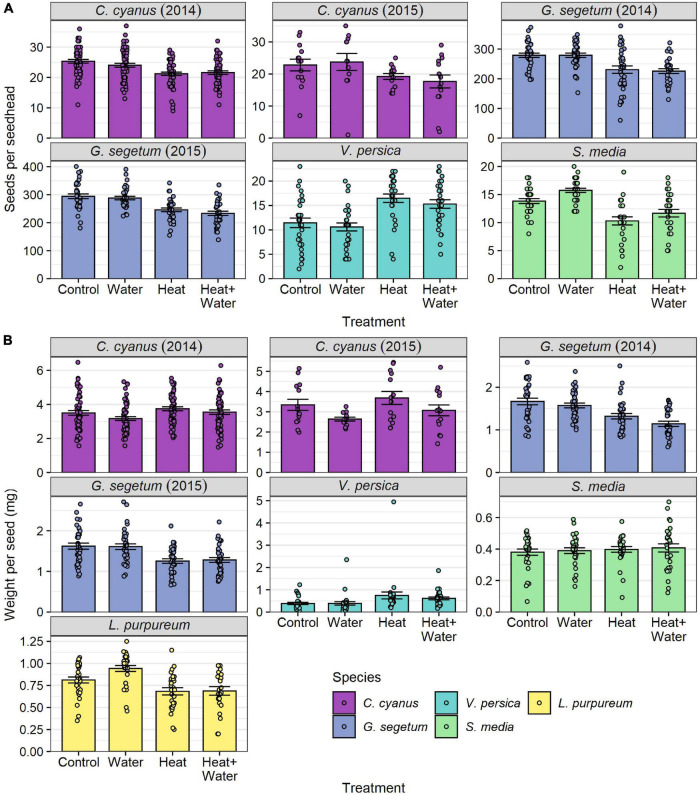
Flowering-plant species responses to treatment for: **(A)** number of seeds per seed head, **(B)** mean seed weight per seed head (mg) (raw values). Bars and error-bars represent mean ± s.e. Points show the individual samples. For *C. cyanus* in 2015, *L. purpureum*, *V. persica*, and *S. media* sampling was restricted by the availability of ripe seed heads, leading to small differences in sample sizes between treatments and a smaller overall sample size for *C. cyanus* in 2015 (see Supplementary Table 6).

All species of wildflower showed significant effects of treatment on seed number per seed head, average seed weight, or both (Table 1, Supplementary Table 6, and Figure 4). *C. cyanus* seed number was lower in the two heated treatments relative to the two unheated ones in both years (precipitation had no effect in either additional water treatment); this was highly significant in 2014 (LRT = 30.125, *p* < 0.001) but non-significant in 2015 (Table 1, Figure 4A, and Supplementary Table 6). Conversely, *C. cyanus* seed weight was slightly higher in the Heat treatment relative to Control in both years, while additional water had the opposite effect causing reduced seed weight, but once again these effects were only significant in 2014 (LRT = 10.895, *p* = 0.012) (Table 1, Figure 4A, and Supplementary Table 6). *G. segetum* showed a consistent pattern of warming reducing both seed number and seed weight in 2014 (number: LRT = 9.186, *p* = 0.027; weight: LRT = 15.543, *p* = 0.001) and 2015 (number: LRT = 23.687, *p* < 0.001; weight: LRT = 18.093, *p* < 0.001) and while precipitation alone had no effects on *G. segetum*, there was a slightly stronger reduction in seed weight in 2014 when warming and precipitation were combined (Figure 4 and Supplementary Table 6). Both warming treatments significantly reduced *L. purpureum* seed weight relative to Control, while additional water increased it (though only in the absence of warming) (LRT = 15.962, *p* = 0.001) (Figure 4B and Supplementary Table 6). *S. media* seed number was significantly reduced in both warming treatments relative to Control (LRT = 20.035, *p* < 0.001) and while precipitation slightly increased seed number, this effect was not significant (Supplementary Table 6 and Figure 4A). *S. media* seed weight was unaffected by any of the treatments (Table 1, Figure 4B, and Supplementary Table 6). *V. persica* was the only species to demonstrate significant increases in both seed number (LRT = 29.646, *p* < 0.001) and seed weight (LRT = 17.323, *p* < 0.001) in response to warming, while precipitation had no significant effects either in the presence or absence of warming (Figure 4 and Supplementary Table 6).

## Discussion

We found that a moderate increase in temperature of 1.5°C caused a significant reduction in the number of flowers and also negatively affected nectar production of some common non-crop plants. Despite finding the experimental treatments having no effect on insect species richness, the community composition was affected, the abundance of visitors was reduced but only in 1 year, and the frequency of visits to individual flowers was increased. The increase in temperature also lead to an increase in flower-visitor network complexity, while consumer-resource asymmetries and structural evenness were unaffected. All but one of the wildflower species examined was negatively affected in terms of seed production, whereas *V. persica* produced more and heavier seeds in the heated treatments. This experiment has yielded the first field-based empirical evidence of *in situ* active-warming impacting arable wildflowers growing within a crop and their interactions with insect pollinators.

### Objective 1: Wildflower Floral Resources

The composition and richness of the floral community were unaffected by the experimental treatments. This is not an unexpected result as the whole experimental area was ploughed before the start of the experiment each year. Studies conducted in sub-alpine and tundra habitats have shown that it can take several years of continuous warming for any changes in vegetation community and richness to be found (Walker et al., 2006; Shi et al., 2015), while experiments in warmer habitats have found no changes even after several years (Price and Waser, 2000; Peñuelas et al., 2007). It is very likely that the timeframe of an annual system that is cut and re-sown every year, is too short to be able to show such floral community changes and any treatment effects are instead likely to be found at the level of the individual organisms. However, being able to discount any long-term effects on community composition means that we can have greater confidence in our outcomes actually representing the impacts of our treatments.

The significant difference in floral abundance is a very striking result that has obvious implications not only for future plant communities via a reduction in fecundity, but also for flower-visiting insects. Our experiment showed that under an increase of 1.5°C there was almost a 40% reduction in floral units throughout the season; this represents a significant decrease in available food for flower visitors. Our findings add to the increasing evidence from a range of plant species that climate warming can cause a reduction in the numbers of flowering plant individuals and/or flowers per plant (Liu et al., 2012; Mu et al., 2015; Takkis et al., 2018). Similarly, the results of our nectar analysis tally with those of other studies that have found increased temperatures cause reduced nectar secretion, and therefore reduce food for flower-visiting insects, but that this response can vary across species (Mu et al., 2015; Takkis et al., 2018; Borghi et al., 2019). Previous floral studies that have incorporated precipitation manipulation into their designs have almost exclusively focussed on reductions (Borghi et al., 2019), therefore our study provides a novel look at the impacts of an increase in precipitation/irrigation in combination with warming. We found that the addition of extra water had no ameliorative effects upon the decreases in floral resources. This points to the underlying mechanism here being one of temperature rather than water stress, which suggests that the negative impacts of climate warming on floral resources could be far worse during periods when water stress is an additional pressure.

### Objective 2: Flower Visitation

Visitor species richness was unaffected by treatment, and while recent research has shown that higher temperatures can lead to lower bee species diversity (Papanikolaou et al., 2017), our findings make sense within the context of our experiment as it was a small scale and lacked any barriers to insect movement. However, we did find an effect of treatment on insect community composition, and a very strong effect of year. This latter finding is unsurprising given that interannual variation in the composition of pollinator communities is extremely common (CaraDonna et al., 2021). Visitor abundance was also significantly reduced in the heated plots, but only in 2014, when the community was dominated by hoverflies, rather than by bees as in 2015. Indeed, most of the overall reduction in abundance seen in 2014 can be attributed to reductions in hoverflies. This suggests that the impact of warming on abundance may have been mediated by the community composition. This difference in community response could be driven by differences in reproductive and foraging behaviours. Bees demonstrate parental care while hoverflies do not, this allows hoverflies to respond differently to resource abundance and habitat structure as they are less spatially restricted than bees (Jauker et al., 2009; Lucas et al., 2017). Therefore, while a difference in the community composition was detected across the treatments, it appears that an overall preference for feeding in the forage-rich unheated plots was only detectable when hoverfly abundance was high. Currently, there are very few published papers that have looked at free-ranging insect responses to experimental climate warming. Berthe et al. (2015) used the same experiment to investigate responses in beetles and also found differing responses between taxonomic groups; the warmed plots contained less-diverse communities dominated by an increased abundance of a small number of Carabidae species, and a reduction in abundance of Staphylinidae species.

The frequency of visits to individual flowers of all species combined, and to those of *G. segetum*, were significantly increased in the heated treatments. This appears to run contrary to evidence indicating that insect visitation is positively affected by floral abundance (Fowler et al., 2016), however, the impacts of simulated climate change can reverse this relationship by affecting other aspects of flower biochemistry and morphology (Borghi et al., 2019). It is likely that the proximity of our experimental plots and the absence of flowers in the surrounding area meant that the whole experimental site represented an attractive foraging patch to insects, but that once they arrived, the drastically reduced floral resources in the heated plots resulted in increased visitation to the flowers within them. It is also possible that reduced nectar volumes in the heated plots could increase the chance of a visitor needing to visit more of the flowers present within them. While increased temperatures could directly impact the foraging behaviour of insect pollinators (Scaven and Rafferty, 2013), it is unlikely to have occurred in our experiment due to the extremely short exposures the insects experienced while foraging in our plots. It is more likely that the increased visitation rate is caused by a far larger change on one side of the equation (floral abundance) than the other (visit abundance).

### Objective 3: Flower-Visitor Networks

Generality was significantly lower in 2015, which can be explained by the lower number of flower species that were recorded that year. The significant effect of year upon weighted connectance is also likely caused by the difference in flower species richness between years; connectance was higher in 2015 when there were fewer flower species present in the plots (Supplementary Table 3) but the same number of insect species (Supplementary Table 4), which makes it more likely that more of the potential interactions were observed.

There was no effect of treatment on network structure (generality or vulnerability), but this is unsurprising given that treatment had no significant effect on species richness for either plants or insects. The significant increase in network complexity (weighted connectance) in the heated treatments means that the insects were visiting a greater proportion of the different flower species present in those plots than in the unheated ones. There is also a trend for higher interaction evenness in the heated treatments. These findings could be explained by the reduction in floral resources in the heated plots causing species to broaden their diets in search of sufficient food, or by a reduction in flower species richness, which would increase the chance of detecting more of the possible interactions. While we found no significant effect of treatment on either diet breadth or plant richness, we did observe a trend for lower values in the heated treatments for both variables. This illustrates the value of using a network approach; if we only looked at these and other variables in isolation then we would miss the cumulative effect of them all combined. It is likely that the observed changes in network complexity are caused by the accruing impact of subtle changes in many aspects of the whole community.

Our findings indicate that flower-visitor network structure is robust to changes in temperature, which supports the conclusions of other studies. In their review, Gérard et al. (2020) concluded that plant-pollinator networks should be resilient to changes in climate due to their nested, asymmetric, and dynamic structure. However, there is also evidence indicating that climate warming can reduce the nestedness of plant-pollinator networks as a result of species loss and diet-breadth shift (Burkle et al., 2013), that phenology is an important determinant of network robustness that is therefore susceptible to climate change (Ramos-Jiliberto et al., 2018), and that climate can directly and indirectly drive network structure via species richness and phenology (Petanidou et al., 2018). This indicates that our experiment did not cause a great enough disturbance to the networks to elicit changes in structure, which is probably because we only directly manipulated the bottom trophic level. Therefore, it is concerning that we found experimental warming increased network complexity despite our bottom-up approach. This suggests that these networks are potentially very sensitive to climate change through the cumulative impact on features such as phenology, floral resources, species richness, and subsequent changes in visitor foraging behaviour.

### Objective 4: Wildflower Seed Set

All five wildflower species were significantly affected by the experimental warming, but the responses differed among species. While three of the species showed clear negative responses to warming (*S. media, L. purpureum*, and *G. segetum*), and one showed clear positive responses (*V. persica*), *C. cyanus* showed a more complex response; producing fewer seeds that were heavier, perhaps demonstrating a compensation for the reduced number. This highlights the complexity of a community-wide response to climate warming. The potential implications are that the plant community could change over time, as species like *S. media* lose out to species like *V. persica*, which are better able to adapt to and capitalise on the new environmental conditions. While precipitation alone had some positive and negative impacts on seed production for some species, additional water did not ameliorate any of the negative impacts of the experimental warming on plant reproduction. While very few studies have investigated seed production in relation to precipitation increases, research using the same experiment to investigate the impacts on wheat also found that additional water could not compensate for the negative effects of the increased temperature (Derocles et al., 2018). This suggests that the negative impacts of climate warming in agro-ecosystems could be severe and difficult to manage.

All five of the species we examined flower from late spring onward, and four of them are generalists in terms of their flower shape, which makes them less susceptible to phenological mismatch (Gérard et al., 2020). Consequently, it is unlikely that our treatments impacted seed production indirectly, except possibly in the case of *L. purpureum*, which has nectaries accessible only to long-tongued insects. Therefore, it seems likely that the impacts we observed on seed set were primarily caused by direct effects on the individual plants themselves. Very few studies have examined the direct impact of increased temperature on wildflower seed set, but there is very strong evidence of negative effects for crop plants (Liu et al., 2016). Jin et al. (2011) found that moderate increases in temperature positively affected *Arabidopsis thaliana* seed weight, but at higher temperatures the impact was negative. Both *A. thaliana* and *V. persica* are common generalist weeds in the United Kingdom (Rose and O’Reilly, 2006), able to flourish in a variety of habitats and when introduced outside of their native range. This adaptable and resilient nature is perhaps why both species are able to cope well under small increases of temperature. In contrast, *G. segetum* and *C. cyanus* are both rare and declining across Europe due to agricultural intensification, as they are restricted to arable land (Sutcliffe and Kay, 2000; Rose and O’Reilly, 2006). Our findings suggest that these rare plants are also threatened by climate change as the negative impacts on seed set (and seed weight for *G. segetum*) have obvious implications for seedling recruitment and long-term population viability. The additional effect of reduced floral abundance on wildflower seed production increases the potential for long-term population and community impacts.

It is particularly interesting that the increased frequency of visits to flowers did not seem to have any beneficial effect on the seed set or seed weight of the flowers that were negatively affected by the treatment. We know that increased visitation can be beneficial for many plants and is linked to increased seed set for some species (Garibaldi et al., 2013). However, there are numerous examples in the literature showing that this relationship is not quite so straight forward, because both insect type and pollinator dependence can be crucial factors in determining how beneficial an insect’s visits to a flower are (Franzén and Larsson, 2009; Lundgren et al., 2013). While the wildflower species we selected rely upon insects for pollen transfer, four of them are self-fertile and can potentially self without the need for pollen vectors (*C. Cyanus* is the exception) (Fitter and Peat, 1994). Therefore, it seems more likely here, that the direct impact of the raised temperature on seed development is having a greater overall effect on seed set than the increased frequency of visits. It is also possible that the lower floral abundance and higher connectivity in the heated plots caused an increase in heterospecific pollen transfer, which could also contribute to decreased seed set. This is potentially very detrimental for some insect-pollinated plants, such as *G. segetum*, as it suggests that climate warming will have negative impacts on their reproduction even if pollinator visitation is increased.

## Conclusion

Our study demonstrates the negative consequences that climate-warming might have on wildflowers and flower-visiting insects in arable farming systems, but it also highlights the need for more experimental field studies considering how climate change may affect species interactions, flowering, and seed set of wildflowers. The considerable inter-annual variation found in the plant and insect communities here also demonstrates the need for longer-term investigations and for greater temporal consideration. We have shown that a 1.5°C increase in temperature can have very large effects upon floral resources, wildflower reproduction, and interaction network complexity, and that such impacts are not offset when water is increased. Our findings also highlight that different species respond to changing climatic conditions very differently, with one species of common generalist weed thriving, while two rare specialist cornfield annuals failed to reproduce as effectively. We simulated representative increases in temperature and precipitation, but not CO_2_, which is an important factor that should also be included in future investigations. Field experiments conducted at larger scales, both in terms of replicate size as well as number, should also be considered a research priority. The focus for climate change research in agricultural landscapes is understandably on yields and food security, but it needs to expand to incorporate a wider range of non-crop organisms and the interactions they provide, including ecosystem services such as insect pollination.

## Data Availability Statement

The datasets presented in this study can be found in online repositories. The names of the repository/repositories and accession number(s) can be found below: Newcastle University Research Repository (https://doi.org/10.25405/data.ncl.17430548).

## Author Contributions

DE conceived the study and secured funding. EM and DE designed the methodology. EM collected and analysed the data and led the writing of the manuscript. Both authors contributed critically to the drafts and gave final approval for publication.

## Conflict of Interest

The authors declare that the research was conducted in the absence of any commercial or financial relationships that could be construed as a potential conflict of interest.

## Publisher’s Note

All claims expressed in this article are solely those of the authors and do not necessarily represent those of their affiliated organizations, or those of the publisher, the editors and the reviewers. Any product that may be evaluated in this article, or claim that may be made by its manufacturer, is not guaranteed or endorsed by the publisher.
