# Supplementary material for: Experimental Climate Warming Reduces Floral Resources and Alters Insect Visitation and Wildflower Seed Set in a Cereal Agro-Ecosystem
Source: Front Plant Sci. 2022 Feb 23;13:826205. doi: 10.3389/fpls.2022.826205 (PMC8905351; doi:10.3389/fpls.2022.826205)
Supplement: Supplementary file 1 [file Data_Sheet_1.pdf]

Supplementary Material

Experimental climate warming reduces floral resources and alters insect visitation and wildflower seed set in a cereal agro-ecosystem

Ellen D. Moss[1,2*], Darren M. Evans[1]

[1]School of Natural and Environmental Sciences, Newcastle University, Newcastle Upon Tyne, UK
[2]School of Biological, Biomedical and Environmental Sciences, University of Hull, Hull, UK

*** Correspondence:**
Dr Ellen Moss
ellen.moss@ncl.ac.uk, ellen.d.moss@gmail.com

1 Materials and Methods

1.1 Experimental design

Average monthly temperatures in 2014 for June, July and August were 16.1, 18.6 and 16.1°C respectively, while the average temperatures for the same months in 2015 were 14.6, 16.8 and 17.4°C respectively.

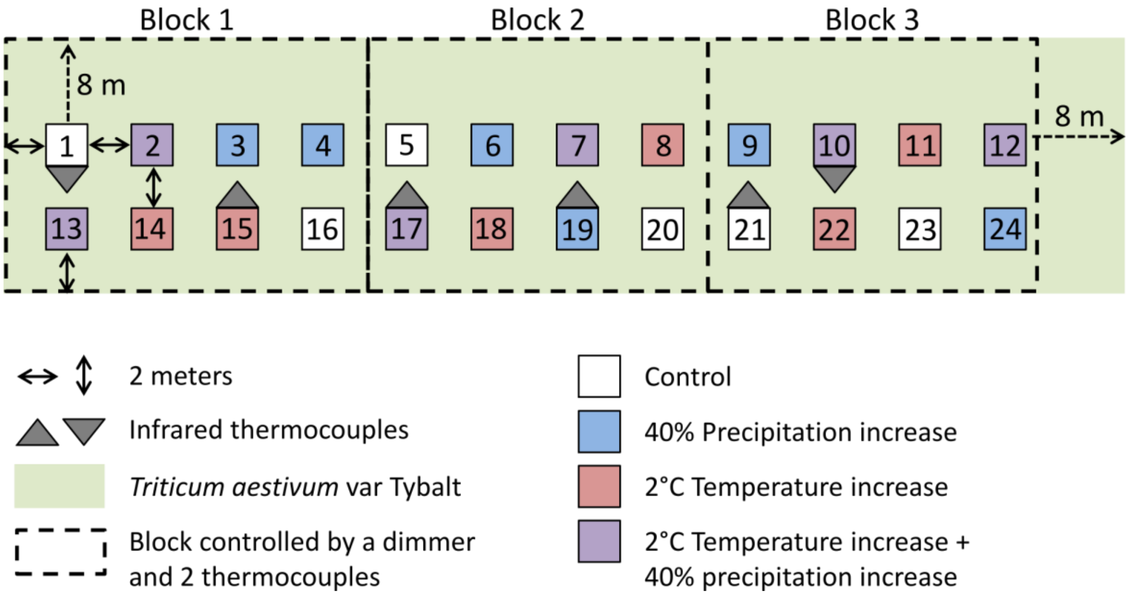

Figure S1**.** Simulated warming experiment layout and design at Stockbridge Technology Centre, Yorkshire, England. Experimental plots were 2 x 2 m in size and were separated by 2 m buffers.

1.2 Wildflowers

All wildflower seeds were purchased from Emorsgate Seeds (http://wildseed.co.uk/home). Wheat was sown on 15/04/14 in the first field season, and wildflowers on 16/04/14. In the second field season wheat was sown on 14/04/15 and wildflowers on 15/04/15.

Table S1. Wildflower species and sowing weights.

Family	Common Name	Scientific Name	Sowing weight per plot (g)
Apiaceae	Upright Hedge Parsley	*Torilis japonica*	2
Asteraceae	Cornflower	*Centaurea cyanus*	0.6
Asteraceae	Corn Marigold	*Glebionis segetum*	0.8
Caryophyllaceae	Corncockle	*Agrostemma githago*	0.2
Fabaceae	Common Vetch	*Vicia sativa*	0.4
Laminaceae	Red deadnettle	*Lamium purpureum*	0.4
Papaveraceae	Common Poppy	*Papaver rhoeas*	0.4
Ranunculaceae	Corn Buttercup	*Ranunculus arvensis*	0.4

1.3 Taxonomic keys

Ball, S.G., 2008. Introduction to the Families of British Diptera. Dipterists Forum.

Chinery, M., 2012. Insects of Britain and Western Europe, 3rd Revised. ed. A&C Black, London.

Collin, J.E., 1961. British Flies (Empididae). Cambridge University Press.

d'Assis Fonseca, E.C.., 1968. Handbooks for the Identification of British Insects. Vol 10 Part 4b. Diptera - Cyclorrhapha Calyptrata, Section (b) Muscidae. Royal Entomological Society, London.

Else, G., Edwards, M., 2018. Handbook of the Bees of the British Isles. The Ray Society, The Natural History Museum, London.

Prys-Jones, O.E., Corbet, S.A., 2011. Bumblebees, 3rd ed. Pelagic Publishing, Exeter, England.

Stubbs, A., Falk, S.J., 2002. British Hoverflies: An Illustrated Identification Guide, 2nd ed. British Entomological & Natural History Society, Reading, England.

Yeo, P., Corbet, S.A., 1995. Solitary Wasps, 2nd Revised. ed. Richmond Publishing Co Ltd, Slough, England.

1.4 Data collection

Abundance of individuals of each plant species was not recorded as it was too difficult to determine without causing damage within the plots, due to the intertwined way that the plants grew. Flowering plant diversity was examined using raw species richness values (cumulative across the season) as the plots were searched exhaustively for flowers during each sample round.

While 5 flower heads were bagged up in preparation for nectar sampling, many flower buds were damaged or failed to open. This resulted in far smaller sample sizes than the target of 30 per treatment and uneven samples between treatments (see Table S3 below).

Flower disc diameter of G. segetum was measured in 2015. After it was determined that the nectaries of this species were too small to sample using the 0.5 microL microcapillary tubes we had available, it was decided to measure the diameter of the floral disc to gain a rough proxy of nectar available via the size of the flower head. The logic being that these are composite flower heads containing many tiny individual inflorescences, a smaller disc means fewer and/or smaller inflorescences and less total nectar per flower head. 10 different flowers were randomly selected per plot, giving sample sizes of 60 per treatment.

While 5 seed heads were collected for each species, for *G. segetum*, only 3 seed heads were processed from each plot. This subsampling was necessary as processing these seed heads was extremely time consuming. In 2015, there were not enough *C. cyanus* plants and seed heads to sample five per plot, so 3 were collected and processed instead. In some cases, sampling of the remaining 3 species was also restricted by the availability of ripe seed heads, leading to slightly uneven sample sizes between treatments (see Table S6 below).

1.5 Data analysis

Table S2. Summary of statistical models created to analyse all plant and insect-visitor response variables. GLM = generalised linear model, MEM = generalised linear (or linear) mixed effects model, LM = linear model, LRT = likelihood ratio test. All link functions were canonical unless specified.

Response Variable	Model	Distribution Family	Predictor(s)	Random Effect(s)	Significance Test
**(1) Floral resources**					
Flower species richness	GLM	Generalised poisson	Treatment + Year	-	LRT
Total floral abundance	GLM	Negative binomial	Treatment + Year	-	LRT
C. cyanus nectar volume*	MEM	Gaussian	Treatment	Plot	LRT
L. purpureum nectar volume	GLM	Gamma	Treatment	-	F
V. persica nectar volume	MEM	Gaussian (log link)	Treatment	Plot	LRT
G. segetum flower diameter**	MEM	Gaussian	Treatment	Plot	LRT
**(2) Visitation**					
Visitor species richness (extrapolated)	GLM	Gamma (log link)	Treatment + Year	-	F
Visitor abundance	GLM	Negative binomial	Treatment * Year	-	LRT
Visits per flower	GLM	Gamma (identity link)	Treatment + Year	-	F
Diet breadth	GLM	Inverse Gaussian	Treatment + Year	-	F
Visits per *C. cyanus* flower	LM	Gaussian	Treatment + Year	-	F
Visits per *G. segetum* flower	GLM	Inverse Gaussian (identity link)	Treatment + Year	-	F
**(3) Networks**					
Weighted connectance	GLM	Beta	Treatment + Year	-	LRT

Generality	GLM	Inverse Gaussian (log link)	Treatment + Year	-	F
Vulnerability	GLM	Gamma (log link)	Treatment + Year	-	F
Interaction evenness	GLM	Beta	Treatment + Year	-	LRT
**(4) Seed set**					
C. cyanus seed number (2014)	MEM	Poisson	Treatment	Date	LRT
C. cyanus seed weight (2014)	MEM	Gaussian	Treatment	Date	LRT
C. cyanus seed number (2015)	GLM	Negative binomial	Treatment	-	LRT
C. cyanus seed weight (2015)	MEM	Gaussian	Treatment	Plot	LRT
G. segetum seed number (2014)	MEM	Poisson	Treatment	Plot + Date	LRT
G. segetum seed weight (2014)	MEM	Gaussian	Treatment	Plot + Date	LRT
G. segetum seed number (2015)	MEM	Negative binomial	Treatment	Plot + Date	LRT
G. segetum seed weight (2015)	MEM	Gaussian	Treatment	Plot	LRT
L. purpureum seed weight	MEM	Gaussian	Treatment	Plot	LRT
V. persica seed number	GLM	Negative binomial	Treatment	-	LRT
V. persica seed weight**	MEM	Gaussian	Treatment	Plot	LRT
S. media seed number	MEM	Generalised poisson	Treatment	Plot	LRT
S. media seed weight***	MEM	Gaussian	Treatment	Plot	LRT

* cube root (^1/3) transformed before analysis

** log transformed before analysis

*** square (^2) transformed before analysis

True count data (positive integer) were analysed with Poisson, negative binomial, or generalised Poisson distributions, the latter two in cases when data were over-dispersed. Positive decimal data were analysed with Gaussian, gamma, or inverse gaussian families. Because the values of Weighted Connectance and Interaction Evenness are bound by 0 and 1, they were analysed with beta distributions.

Link function was primarily selected based upon two factors: firstly, suitability for the data; where an extensive variable was not transformed, a transformative link function was used (usually a log link). And secondly, fit of the model; link functions were left as the canonical except when another provided a substantially better fit.

Model fit was assessed by examining simulated residuals created using the DHARMa R package (Hartig, 2021). This allowed easy checking of the fit of the distribution family (and link function), and for over/under dispersion.

Data were transformed when all distribution and link options produced poor fitting models. Transformed variables were then analysed with a Gaussian distribution. Multiple transformations were tried and the one that provided the best fitting model was selected.

If the mixed effects models were found to be singular, then the random terms were removed if their variance was 0 (or nearly 0). This was the case for *Lamium purpureum* nectar volume, *Centaurea cyanus* seed number (2015), and *Veronica persica* seed number.

‘Block’ (see Figure S1) was not included as a random effect in the models due to the small sample sizes limiting the number of random effects that could feasibly be included. The adjacency of the blocks, close proximity of the plots within the blocks, and relatively few samples from each plot, meant that ‘plot’ alone was able to account for any spatial variation or patterning.

Significance of the Treatment:Year interaction in the LM and GLM models was determined using sequential tests (Type I) via the ‘anova()’ function, using either “Chisq” or “F” depending upon the distribution (Chisq: Poisson, negative binomial, generalised Poisson; F: Gaussian, gamma, inverse gaussian). Use of the ‘anova()’ function was not compatible with those models fit with the ‘glmmTMB’ package (Brooks et al., 2017) (Floral abundance, Weighted connectance, and Interaction evenness), so marginal tests (Type II) via the ‘drop1’ function were used instead. Where the interaction was not significant, significance of the two main effects (Treatment and Year) was determined via marginal tests also using the ‘drop1’ function, again using “Chisq” or “F” depending upon the distribution. Significance of Treatment in the mixed effects models was also determined via marginal tests using the ‘drop1’ function. It is worth noting that both ‘anova(model, test = “Chisq”)’ and ‘drop1(model, test = “Chisq”)’ run likelihood ratio tests, which means in reality these tests were all either F tests or LRTs (Table S2).

PERMANOVA analysis of community composition was conducted using the ‘adonis2’ function of the vegan package in R (Oksanen et al., 2020). Sequential tests were used to assess the interaction term, and as these were non-significant for both plants and insects, the models were re-run without the interaction using marginal tests. For flowering plant species, the ‘bray’ method was used, and binary was set to ‘TRUE’ as numbers of individuals had not been recorded. For the insect species, the ‘chao’ method was used as the species accumulation curves had not demonstrated sampling completeness.

The degrees of freedom for the F and LRT (likelihood ratio) tests were the same for each element: Treatment = 3, Year = 1, Treatment:Year interaction = 3.

Frequency of visits to plant species other than C. cyanus and G. segetum could not be analysed due to insufficient visit data.

Brooks, M. E., Kristensen, K., Benthem, K. J. van, Magnusson, A., Berg, C. W., Nielsen, A., et al. (2017). glmmTMB Balances Speed and Flexibility Among Packages for Zero-inflated Generalized Linear Mixed Modeling. *The R Journal* 9, 378–400.

Hartig, F. (2021). *DHARMa: Residual Diagnostics for Hierarchical (Multi-Level / Mixed) Regression Models. R package version 0.4.1.* Available at: http://florianhartig.github.io/DHARMa/.

Oksanen, J., Blanchet, F. G., Kindt, R., Legendre, P., Minchin, P. R., O’Hara, R. B., et al. (2020). *vegan: Community Ecology Package. R Package version 2.5-7.* Available at: https://cran.r-project.org/web/packages/vegan/index.html.

2 Results

2.1 Flower data

Table S3. Effect of treatment and year on all flowering plant variables. Mean values for each factor level are of the raw data, except where it was transformed before analysis. For floral richness and abundance, estimated coefficients are taken from models without the interaction term. Estimates for Heat, Heat+Water, and Water are relative to Control (this was the intercept in all the models), and those of Year 2 are relative to Year 1. Mean values and estimates are given ± standard errors. Significant *p* values (p<0.05) are italicised. Sampling for floral richness and abundance was at the plot level (1 value per plot per year, in total 12 per treatment and 24 per year). For the nectar variables, multiple samples were taken from each plot, the target number was 5 per plot but sampling was restricted by the loss of flowers after bagging in many cases.

Variable	*n*	Factor Level	Mean	Estimate	Statistic	Value	*p*
Floral richness	12	Control	12.250 ± 0.9857	2.6803 ± 0.0460	z	58.29	*<0.001*
	12	Water	12.000 ± 0.6396	-0.0260 ± 0.0615	z	-0.42	0.672
	12	Heat	10.333 ± 1.0684	-0.1306 ± 0.0623	z	-2.1	*0.036*
	12	Heat+Water	11.667 ± 0.7914	-0.0354 ± 0.0611	z	-0.58	0.562
		Year 2	9.1250 ± 0.4007	-0.4141 ± 0.0446	z	-9.29	*<0.001*
		Year 1	14.000 ± 0.3510	- -	-	-	-
Total floral abundance	12	Control	1648.33 ± 143.25	7.2555 ± 0.1125	z	64.479	*<0.001*
	12	Water	1631.75 ± 151.55	-0.0383 ± 0.1423	z	-0.269	0.788
	12	Heat	1029.75 ± 101.16	-0.4922 ± 0.1424	z	-3.457	*<0.001*
	12	Heat+Water	1056.17 ± 165.46	-0.4559 ± 0.1424	z	-3.202	*0.001*
		Year 2	1544.42 ± 118.82	0.3106 ± 0.1007	z	3.084	*0.002*
		Year 1	1138.58 ± 97.241	- -	-	-	-
C. cyanus nectar volume*	12	Control	0.5816 ± 0.0251	0.5711 ± 0.0452	t	12.628	-
	17	Water	0.6408 ± 0.0251	0.0662 ± 0.0639	t	1.035	-
	19	Heat	0.5365 ± 0.0297	-0.0285 ± 0.0613	t	-0.466	-
	13	Heat+Water	0.5101 ± 0.0399	-0.0575 ± 0.0654	t	-0.878	-
L. purpureum nectar volume	12	Control	0.3758 ± 0.0399	2.6610 ± 0.3660	t	*7.271*	*<0.001*
	13	Water	0.3472 ± 0.0327	0.2194 ± 0.4150	t	0.415	0.681
	5	Heat	0.1032 ± 0.0389	7.0281 ± 2.0967	t	3.352	*0.002*
	4	Heat+Water	0.2578 ± 0.0781	1.2181 ± 0.9940	t	1.226	0.230
V. persica nectar volume	12	Control	0.1426 ± 0.0168	-2.1374 ± 0.2358	t	-9.063	*<0.001*
	11	Water	0.1269 ± 0.0167	-0.0167 ± 0.3027	t	-0.055	0.956
	8	Heat	0.0504 ± 0.0093	-0.9091 ± 0.3316	t	-2.741	*0.006*
	8	Heat+Water	0.0379 ± 0.0057	-1.1511 ± 0.3643	t	-3.160	*0.002*
G. segetum flower disc diameter (mm)**	60	Control	2.6490 ± 0.0158	2.6490 ± 0.0334	t	79.360	-
	60	Water	2.7138 ± 0.0155	0.0648 ± 0.0472	t	1.373	-
	60	Heat	2.5591 ± 0.0152	-0.0899 ± 0.0472	t	-1.904	-
	60	Heat+Water	2.5746 ± 0.0203	-0.0744 ± 0.0472	t	-1.575	-

* cube root transformed before analysis

** log transformed before analysis

G. segetum flower disc diameter was analysed using a mixed effect model, treatment had a significant effect (LRT=12.568, p=0.006).

2.2 Insect data

Table S4. Effect of treatment and year on all insect-visitor variables. Mean values for each factor level are of the raw data, those for each treatment level are for both years combined except for visitor abundance, where they are for each treatment in each year separately. Estimated coefficients are taken from models without the interaction term unless it was significant (visitor abundance). Estimates for Heat, Heat+Water, Water and Year 2 are relative to Control and Year 1 (this was the intercept in all the models). Mean values and estimates are given ± standard errors. Significant *p* values (*p*<0.05) are italicised. Sampling for all variables was at the plot level (1 value per plot per year, in total 12 per treatment and 24 per year).

Variable	*n*	Factor Level	Mean	Estimate	Statistic	Value	*p*
Visitor species richness (extrapolated)	12	Control	36.128 ± 4.5008	3.5803 ± 0.1625	t	22.036	*<0.001*
	12	Water	42.269 ± 6.0873	0.1566 ± 0.2055	t	0.762	0.45
	12	Heat	36.955 ± 4.8247	0.0237 ± 0.2055	t	0.115	0.909
	12	Heat+Water	35.727 ± 6.1741	-0.0121 ± 0.2055	t	-0.059	0.953
		Year 2	38.045 ± 4.7703	0.0137 ± 0.1453	t	0.094	0.925
		Year 1	37.495 ± 2.4956	- -	-	-	-
Visitor abundance	12	Control	85.500 ± 5.1559	4.4490 ± 0.0846	z	52.559	*<0.001*
	12	Water	75.167 ± 4.8471	-0.1288 ± 0.1208	z	-1.066	0.286
	12	Heat	59.500 ± 8.1925	-0.3625 ± 0.1232	z	-2.943	*0.003*
	12	Heat+Water	61.000 ± 5.8023	-0.3376 ± 0.1229	z	-2.747	*0.006*
		Year 2	85.500 ± 8.2452	-2.13E-15 ± 0.1197	z	0.000	1.000
		Water: Year 2	89.833 ± 7.0020	0.1782 ± 0.1698	z	1.050	0.294
		Heat: Year 2	97.667 ± 8.3533	0.4956 ± 0.1711	z	2.897	*0.004*
		Heat+Water: Year 2	92.833 ± 10.378	0.4199 ± 0.1711	z	2.454	*0.014*
Visits per flower (all plant species)	12	Control	0.0553 ± 0.0040	0.0581 ± 0.0062	t	*9.406*	*<0.001*
	12	Water	0.0540 ± 0.0046	-0.0019 ± 0.0072	t	-0.266	0.792
	12	Heat	0.0794 ± 0.0077	0.0248 ± 0.0092	t	2.706	*0.010*
	12	Heat+Water	0.0863 ± 0.0102	0.0315 ± 0.0097	t	3.253	*0.002*
		Year 2	0.0683 ± 0.0063	-0.0057 ± 0.0061	t	-0.943	0.351
		Year 1	0.069 ± 0.005	- -	-	-	-
Diet breadth	12	Control	1.3034 ± 0.0471	0.5198 ± 0.0421	t	12.335	*<0.001*
	12	Water	1.3436 ± 0.0498	-0.0343 ± 0.0538	t	-0.637	0.527
	12	Heat	1.2277 ± 0.0452	0.0740 ± 0.0577	t	1.282	0.2067
	12	Heat+Water	1.3076 ± 0.0529	-0.0037 ± 0.0549	t	-0.068	0.946
		Year 2	1.2131 ± 0.0302	0.1524 ± 0.0399	t	3.816	*<0.001*
		Year 1	1.3780 ± 0.0305	- -	-	-	-

Continued from previous page

**Variable**	***n***	**Factor Level**	**Mean**	**Estimate**	**Statistic**	**Value**	***p***
Visits per *C. cyanus* flower	12	Control	0.1922 ± 0.0508	0.1720 ± 0.0389	t	4.418	*<0.001*
	12	Water	0.1479 ± 0.0249	-0.0443 ± 0.0501	t	-0.885	0.381
	12	Heat	0.1662 ± 0.0222	-0.0260 ± 0.0501	t	-0.520	0.606
	12	Heat+Water	0.2300 ± 0.0373	0.0377 ± 0.0501	t	0.753	0.456
		Year 1	0.1638 ± 0.0162	0.1720 ± 0.0389	t	1.251	0.218
		Year 2	0.2084 ± 0.0338	- -	-	-	-
Visits per *G. segetum* flower	12	Control	0.0938 ± 0.0102	0.0960 ± 0.0104	t	9.229	*<0.001*
	12	Water	0.0912 ± 0.0073	-0.0023 ± 0.0122	t	-0.187	0.852
	12	Heat	0.1285 ± 0.0129	0.0334 ± 0.0164	t	2.04	*0.048*
	12	Heat+Water	0.1407 ± 0.0158	0.0461 ± 0.0182	t	2.53	*0.015*
		Year 2	0.1058 ± 0.0067	-0.0038 ± 0.0105	t	-0.365	0.717
		Year 1	0.1213 ± 0.0113	- -	-	-	-

2.3 Network data

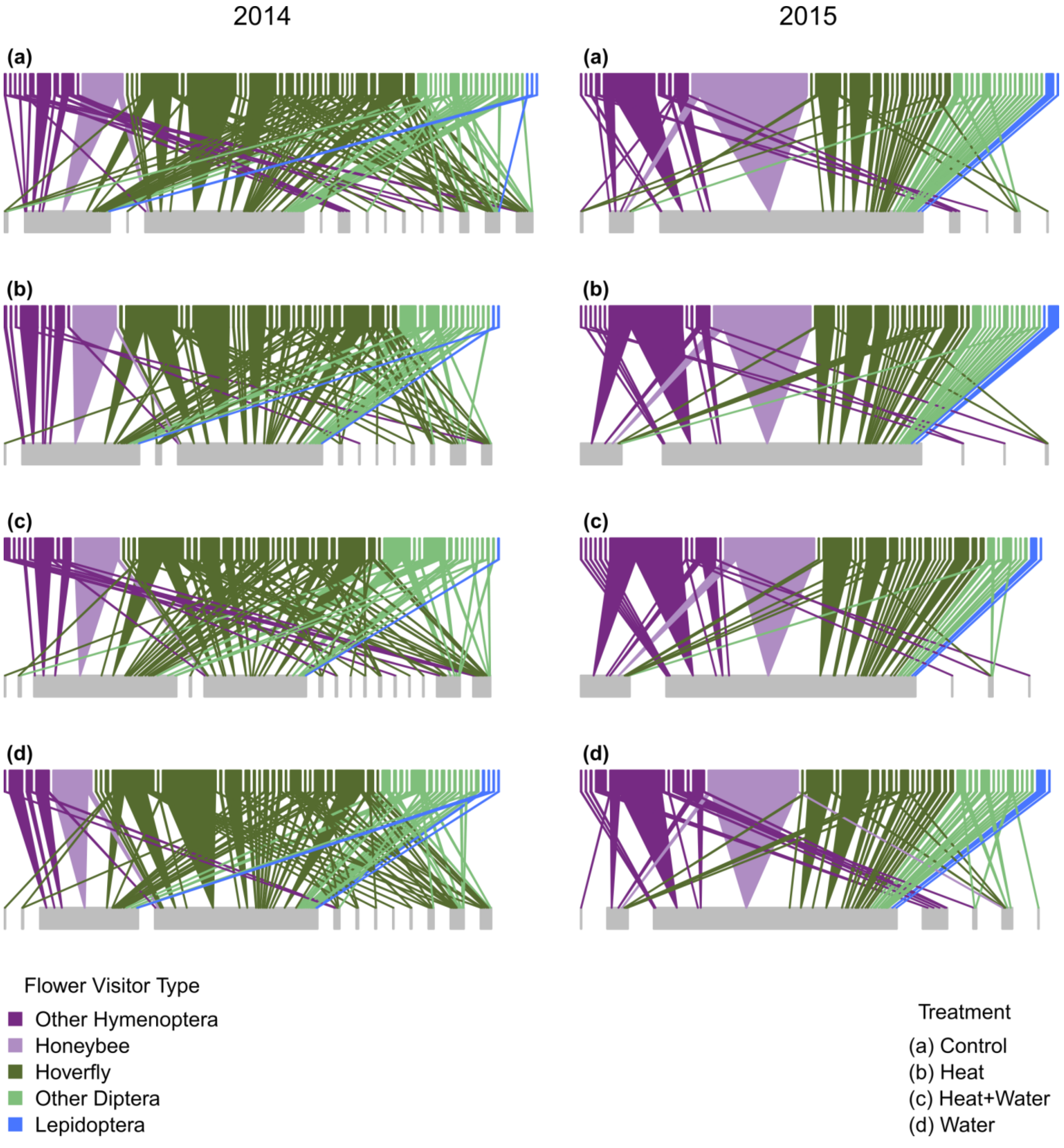

Figure S2. Species interaction networks for each treatment in both sampling years, showing the different flower visitors (top) and plants (bottom). Different flower-visitor types are represented by different colours. Width of species rectangles represents total number of interactions that species made and width of connections between the trophic levels represents the frequency of that particular plant-insect interaction pairing.

Table S5. Effect of treatment and year on all network descriptor variables. Mean values for each factor level are of the raw data. Estimated coefficients are taken from models without the interaction term. Estimates for Heat, Heat+Water, Water and Year 2 are relative to Control and Year 1 (this was the intercept in all the models). Mean values and estimates are given ± standard errors. Significant *p* values (p<0.05) are italicised. Sampling for all variables was at the plot level (1 value per plot per year, in total 12 per treatment and 24 per year).

**Variable**	***n***	**Treatment**	**Mean**	**Estimate**	**Statistic**	**Value**	***p***
Weighted connectance	12	Control	0.1675 ± 0.0121	-1.7575 ± 0.0747	z	-23.696	*<0.001*
	12	Water	0.1750 ± 0.0070	0.0562 ± 0.0925	z	0.607	0.544
	12	Heat	0.2052 ± 0.0166	0.2340 ± 0.0902	z	2.595	*0.009*
	12	Heat+Water	0.2131 ± 0.0125	0.2974 ± 0.0895	z	3.327	*0.001*
		Year 2	0.2135 ± 0.0090	0.3015 ± 0.0633	z	4.757	*<0.001*
		Year 1	0.1670 ± 0.0073	- -	-	-	-
Generality	12	Control	1.4110 ± 0.0732	0.4225 ± 0.0535	t	7.892	*<0.001*
	12	Water	1.4527 ± 0.0677	0.0254 ± 0.0668	t	0.379	0.706
	12	Heat	1.4343 ± 0.0833	0.0154 ± 0.0667	t	0.230	0.819
	12	Heat+Water	1.4444 ± 0.0730	0.0302 ± 0.0669	t	0.451	0.655
		Year 2	1.3184 ± 0.0470	-0.1640 ± 0.0475	t	-3.450	*0.001*
		Year 1	1.5528 ± 0.0439	- -	-	-	-
Vulnerability	12	Control	7.1448 ± 0.4275	1.9417 ± 0.0640	t	30.325	*<0.001*
	12	Water	7.5102 ± 0.3286	0.0498 ± 0.0810	t	0.615	0.542
	12	Heat	7.5470 ± 0.4358	0.0535 ± 0.0810	t	0.661	0.512
	12	Heat+Water	7.5686 ± 0.4996	0.0562 ± 0.0810	t	0.693	0.492
		Year 2	7.6320 ± 0.3319	0.0501 ± 0.0573	t	0.875	0.387
		Year 1	7.2533 ± 0.2529	- -	-	-	-
Interaction evenness	12	Control	0.5870 ± 0.0203	0.3987 ± 0.0816	z	4.898	*<0.001*
	12	Water	0.6055 ± 0.0110	0.0715 ± 0.1030	z	0.695	0.487
	12	Heat	0.6531 ± 0.0234	0.2931 ± 0.1045	z	2.806	*0.005*
	12	Heat+Water	0.6459 ± 0.0148	0.2488 ± 0.1042	z	2.387	*0.017*
		Year 2	0.6108 ± 0.0165	-0.0942 ± 0.0739	z	-1.275	0.202
		Year 1	0.6349 ± 0.0096	- -	-	-	-

2.4 Seed data

Table S6. Effect of treatment on all wildflower seed variables. Mean values for each factor level are of the raw data, except where it was transformed before analysis. Coefficient estimates for Heat, Heat+Water and Water are relative to Control, which was the intercept in all the models. Mean values and estimates are given ± standard errors. Significant *p* values (*p*<0.05) are italicised. Multiple samples were taken from each plot, the target number was 5 per plot but sampling was restricted by availability of ripe seed heads in some cases. There were two sampling events for *G. segetum* in both years, and for *C. cyanus* in 2014, to account for the prolonged flowering periods observed in these two species (this accounts for the higher sample sizes for *C. cyanus* in 2014).

Variable	*n*	Treatment	Mean	Estimate	Statistic	Value	*p*
C. cyanus seed number (2014)	60	Control	25.333 ± 0.5859	3.2312 ± 0.0394	z	82.071	*<0.001*
	60	Water	24.017 ± 0.6437	-0.0534 ± 0.0368	z	-1.452	0.147
	60	Heat	21.217 ± 0.5519	-0.1773 ± 0.0380	z	-4.668	*<0.001*
	60	Heat+Water	21.600 ± 0.5666	-0.1594 ± 0.0378	z	-4.217	*<0.001*
C. cyanus seed weight (2014)	60	Control	3.4990 ± 0.1371	3.4990 ± 0.4243	t	8.246	-
	60	Water	3.1690 ± 0.1104	-0.3300 ± 0.1674	t	-1.971	-
	60	Heat	3.7418 ± 0.1145	0.2429 ± 0.1674	t	1.451	-
	60	Heat+Water	3.5445 ± 0.1323	0.0456 ± 0.1674	t	0.272	-
G. segetum seed number (2014)	36	Control	279.08 ± 7.1160	5.6307 ± 0.0649	z	86.746	*<0.001*
	36	Water	279.08 ± 7.2852	0.0002 ± 0.0917	z	0.002	0.999
	36	Heat	230.64 ± 12.294	-0.2276 ± 0.0918	z	-2.478	*0.013*
	30	Heat+Water	225.73 ± 8.1813	-0.2171 ± 0.0963	z	-2.254	*0.024*
G. segetum seed weight (2014)	36	Control	1.6681 ± 0.0772	1.6681 ± 0.1001	t	16.668	-
	36	Water	1.5725 ± 0.0557	-0.0956 ± 0.1303	t	-0.733	-
	36	Heat	1.3194 ± 0.0637	-0.3487 ± 0.1303	t	-2.675	-
	30	Heat+Water	1.1424 ± 0.0618	-0.5257 ± 0.1367	t	-3.846	-
C. cyanus seed number (2015)	14	Control	22.786 ± 1.8283	3.1261 ± 0.0988	z	31.64	*<0.001*
	12	Water	23.750 ± 2.6490	0.0415 ± 0.1449	z	0.286	0.775
	14	Heat	19.214 ± 0.9089	-0.1705 ± 0.1418	z	-1.202	0.229
	15	Heat+Water	17.667 ± 2.0087	-0.2545 ± 0.1404	z	-1.812	0.07
C. cyanus seed weight (2015)	14	Control	3.3440 ± 0.2736	3.4218 ± 0.3318	t	10.312	-
	12	Water	2.6383 ± 0.0952	-0.7835 ± 0.5048	t	-1.552	-
	14	Heat	3.6848 ± 0.3202	0.2195 ± 0.4792	t	0.458	-
	15	Heat+Water	3.0714 ± 0.2660	-0.3504 ± 0.4752	t	-0.737	-
G. segetum seed number (2015)	36	Control	294.33 ± 8.2588	5.6833 ± 0.0335	z	169.92	*<0.001*
	36	Water	288.11 ± 6.2537	-0.0207 ± 0.0440	z	-0.47	0.638
	36	Heat	245.97 ± 6.6079	-0.1788 ± 0.0442	z	-4.048	*<0.001*
	36	Heat+Water	233.56 ± 7.5796	-0.2332 ± 0.0443	z	-5.269	*<0.001*
G. segetum seed weight (2015)	36	Control	1.6212 ± 0.0744	1.6212 ± 0.0735	t	22.057	-
	36	Water	1.6090 ± 0.0694	-0.0122 ± 0.1040	t	-0.117	-
	36	Heat	1.2535 ± 0.0559	-0.3677 ± 0.1040	t	-3.537	-
	36	Heat+Water	1.2806 ± 0.0596	-0.3406 ± 0.1040	t	-3.277	-

Continued from previous page

Variable	*n*	Treatment	Mean	Estimate	Statistic	Value	*p*
L. purpureum Seed Weight	30	Control	0.8117 ± 0.0340	0.8117 ± 0.0465	t	17.459	-
	30	Water	0.9423 ± 0.0331	0.1306 ± 0.0658	t	1.987	-
	27	Heat	0.6830 ± 0.0407	-0.1247 ± 0.0669	t	-1.864	-
	23	Heat+Water	0.6873 ± 0.0482	-0.1231 ± 0.0700	t	-1.758	-
V. persica Seed Number	30	Control	11.433 ± 0.9811	2.4365 ± 0.0710	z	34.309	*<0.001*
	30	Water	10.600 ± 0.8089	-0.0757 ± 0.1016	z	-0.745	0.456
	29	Heat	16.483 ± 0.8576	0.3658 ± 0.0966	z	3.785	*<0.001*
	30	Heat+Water	15.300 ± 0.8725	0.2913 ± 0.0967	z	3.013	*0.002*
V. persica Seed Weight*	30	Control	-1.1790 ± 0.1242	-1.1790 ± 0.1447	t	-8.149	-
	30	Water	-1.2606 ± 0.1420	-0.0816 ± 0.2046	t	-0.399	-
	29	Heat	-0.5107 ± 0.1040	0.6756 ± 0.2057	t	3.284	-
	30	Heat+Water	-0.6204 ± 0.0951	0.5586 ± 0.2046	t	2.730	-
S. media Seed Number	30	Control	13.833 ± 0.4397	2.6203 ± 0.0527	z	49.76	*<0.001*
	30	Water	15.733 ± 0.3649	0.1264 ± 0.0732	z	1.73	0.0843
	27	Heat	10.296 ± 0.7276	-0.2774 ± 0.0792	z	-3.5	*<0.001*
	30	Heat+Water	11.667 ± 0.6667	-0.1621 ± 0.0762	z	-2.13	*0.0335*
S. media Seed Weight**	30	Control	0.1560 ± 0.0125	0.1560 ± 0.0201	t	7.753	-
	30	Water	0.1614 ± 0.0136	0.0054 ± 0.0285	t	0.19	-
	27	Heat	0.1667 ± 0.0124	0.0105 ± 0.0289	t	0.363	-
	30	Heat+Water	0.1855 ± 0.0214	0.0296 ± 0.0285	t	1.04	-

* log transformed before analysis

** square (^2) transformed before analysis

2.5 Community composition

Table S7. Effect of treatment and year on the flowering plant and insect community compositions from PERMANOVA models (d.f = 1 for year, 3 for treatment, 3 for treatment:year interaction). Treatment and year *p* values are taken from models without the interaction term. Significant *p* values ($p<0.05$) are italicised. Sampling for both variables was at the plot level (1 value per plot per year, in total 12 per treatment and 24 per year).

		Treatment		Year		Interaction	
**Response variable**	***n***	**F value**	***p***	**F value**	***p***	**F value**	***p***
Flowering plant community	48	1.718	0.074	20.549	*0.001*	1.143	0.365
Insect community (extrapolated)	48	4.031	*0.004*	44.261	*0.001*	0.545	0.735
